# Alterations in the Intestinal Microbiome and Metabolic Profile of British Shorthair Kittens Fed with Milk Replacer

**DOI:** 10.3390/ani14162346

**Published:** 2024-08-14

**Authors:** Cheng Wang, Qi Zhu, Yinan Li, Jiaxing Guo, Lian Li

**Affiliations:** College of Animal Science and Technology, Nanjing Agricultural University, Nanjing 210095, China

**Keywords:** cat, milk replacer, gut microbiota, metabolomics, metabolic pathways, microbiome

## Abstract

**Simple Summary:**

The nutritional needs of kittens are normally met by the queen, but sometimes unfavorable events such as maternal behavior, blood incompatibility and maternal agalactia occur that make it necessary for breeders to use milk replacer for feeding. As the breeding of domestic cats grows, so does the demand for specialized kitten formula. The aim of this study was to investigate the effects of an experimental milk replacer (EMR) on growth, gut microbiology, immune antioxidant capacity and nutrient metabolism in kittens. The results revealed that this EMR not only increases the growth rate of kittens in the late lactation period but also modified the composition of the gut microbiome, improved antioxidant capacity and activated lipid metabolic pathways. These insights are pivotal for optimizing milk replacers for kittens, thereby addressing challenges in feline neonatal nutrition.

**Abstract:**

With the rising popularity of pet cats as companion animals, the survival of newborn kittens is often threatened by factors such as inadequate nursing, maternal behavior and blood incompatibility. These challenges require the use of milk replacers for nurturing. To investigate the effects that feeding kittens with an experimental milk replacer (EMR) have on growth and development, intestinal microbiota, immune response and nutrient metabolism, 12 British shorthair kittens were randomly divided into two groups after nursing for the first week of life. Kittens were fed queen’s milk or EMR, whereby kittens fed queen’s milk served as the control (CON) group. The findings revealed that the CON group exhibited superoxide dismutase (SOD) activity and total antioxidant capacity (T-AOC) (*p* < 0.01) on day 7. However, the EMR group had better growth performance during the later stage of the experiment (*p* < 0.05); the immunocompetence and antioxidant capacity of the EMR group were not significantly different from those of the CON group in the middle and late stages of the experiment, and the mean values of all the indexes were slightly better than those of the control group. Sequencing of the 16S rRNA gene in microbiota demonstrated that EMR increased the colonization of bacterial genera, including *Lachnospiraceae*, *Enterococcus*, *Rothia* and *Ligilactobacillus*. Compared to the CON group, acetate acid (*p* < 0.05), propionate acid (*p* < 0.01) and total SCFAs (*p* < 0.01) in the EMR group were significantly increased. Moreover, the intake of the EMR resulted in the production of distinct metabolites implicated in the metabolism of lipids and amino acids, among other nutrients, thus invigorating the associated metabolic pathways. These results elucidate the impact of administering a milk replacer on gastrointestinal health and nutrient assimilation in kittens. The study provides insights into the use of milk powder alternatives and sets the stage for future research on the formulation and effectiveness of kitten milk replacers.

## 1. Introduction

With the growing integration of pets into human society, domestic cats have risen to prominence as the most cherished companions. Estimates indicate a global feline population of 600 million to one billion, inclusive of pet cats [1]. However, data indicate that the mortality rate for neonatal cats, not counting stillbirths, stands at 8.2% [2]. As the number of domestic cats increases, so does the number of lactation problems that occur, creating a demand for milk replacers. Factors such as maternal behavioral issues, neonatal isoerythrolysis (NI) and maternal agalactia or hypogalactia significantly impact the survival rates of newborn kittens [3]. Females giving birth for the first time or experiencing stress are likely to develop rejecting or aggressive behavior toward their newborns and are more prone to nursing deficiencies [4,5]. Moreover, domestic cats usually have three groups of blood types (A, B and AB), and they produce natural alloantibodies which can neutralize the erythrocytes of another group. NI often occurs in litters where kittens with blood group A are born to a mother with blood group B [6]. These factors, combined with various peripartum pathologies, compel the pursuit of kitten milk replacers to ensure the survival of these young felines.

Breastfeeding is the paramount method of infant feeding, offering a plethora of nutrients and a foundational immune defense for newborns [7]. Colostrum enriched with proteins and lipids serves as the primary energy source, with these components contributing 50% and 40% of its total energy, respectively [8]. As kittens progress through lactation, lactose in queen’s milk emerges as their principal sugar source. Vitamins A, E and D are abundant in colostrum, playing a pivotal role in promoting growth, enhancing nutrient absorption and bolstering the immunity of young kittens [9]. Colostrum is also characterized by its high concentration of immunoglobulins, notably IgG, facilitating the passive transfer of maternal antibodies and fostering both systemic and local immunity in newborns [9,10]. While feline colostrum boasts an energy content of 1300 kcal/L, milk replacers vary widely, ranging from 500 to 1500 kcal/L, and are generally deficient in fat and protein compared to natural queen’s milk [11,12]. Although some milk replacers incorporate immune proteins such as whey protein and lactoferrin, they fall short in replicating the unique immune-enhancing elements inherent in the mother’s milk.

Dietary inputs significantly influence the development of the gut and its bacterial composition in young mammals. The onset of diarrhea, often accompanied by alterations in the composition of intestinal bacteria and gut activity, is a common consequence of early or abrupt weaning [13]. Previous research indicates that administering even small quantities of formula milk to infants can precipitate significant shifts in gastrointestinal (GI) bacteria [14]. Formula-fed infants exhibit a lower density of lactic acid-producing bacteria in their feces than their breastfed counterparts [15]. Research in mouse models suggests that formula feeding disrupts initial microbial colonization in neonates, leading to reduced α-diversity within the intestinal microbiota. Furthermore, a unique β-diversity pattern emerges, characterized by an increased presence of the genera *Enterobacteriaceae* and *Enterococcus* in the ileum [16]. Regarding short-chain fatty acids (SCFAs), previous studies have shown the overall fecal concentration of SCFAs was comparable across both feeding methods. Notably, breastfed preterm infants had elevated levels of propionate and comparatively reduced levels of acetate in their feces [17]. However, the influence of milk replacers on intestinal microbiota in domestic cats requires further research to be clearly delineated.

This study was conducted to evaluate the effects of kitten milk replacer on the growth performance, intestinal microbial structure, metabolic profile and immuno-antioxidant capacity of weaned kittens. It serves as a reference and basis for the production and use of kitten milk replacer.

## 2. Materials and Methods

### 2.1. Experimental Milk Replacers

In this study, the experimental milk replacer (EMR) used was kitten formula milk powder manufactured by IN^®^ (Nanjing, China). According to the product label, this milk is suitable for orphaned or undersized kittens or those that receive insufficient milk from the queen. The dietary composition and nutrient content of the kitten formula as derived from the manufacturer’s product description is shown in Table 1. The kitten formula milk was prepared and administrated in accordance with the manufacturer’s instructions (Table 2).

### 2.2. Experimental Design

Three litters of cats were obtained for this study, all living in the same cattery environment. The kittens were born after a one-week period of acquired immunization, during which all kittens lived with queens and were nursed. One week later, 12 kittens were selected from all 3 litters based on mother, sex and body weight (BW), and the kittens were divided into two groups of six kittens each using a randomized grouping process designed to minimize differences between groups; this was defined as day 0 of the experimental period. To reduce intergroup differences, each litter of kittens was assigned to different groups (Table 3). Animal experiments were approved by the Laboratory Animal Welfare Ethics Review Committee of Nanjing Agricultural University (SYXK (SU) 2017-0027).

One group continued to live with the queens, and these kittens were nursed by their mothers, while the other group started to receive EMR feedings. Both groups of kittens lived in the same cattery but were separated into different cages. The specific dilution of the EMR was performed as follows: before preparing the milk, the operator would disinfect their hands and utensils as required. The water was then boiled and cooled to 70 °C, and the milk was diluted at a ratio of 30 mL of water for every 5 g of powdered milk. After that, the milk was cooled down to 37 °C and the appropriate teat was used for nursing. Table 2 shows the amount of powdered milk fed, which may have deviated slightly in practice. The whole trial lasted for 28 days. BW was recorded every seven days, and fresh fecal samples were collected on days 7, 14 and 28.

### 2.3. Fecal Microbiota Analysis

DNA extraction and sequencing were completed by Wuhan Genecreate Biological Engineering Co., Ltd. (Wuhan, China). according to previous experience and methods [18]. PCR amplification was performed using Pfu high-fidelity DNA polymerase from Beijing TransGen Biotech Co., Ltd. (Beijing, China). The V3–V4 region of the bacterial 16S rRNA gene was amplified via PCR using primers 341F and 806R. Magnetic bead purification of amplification products was conducted using VAHTSTM DNA Clean Beads (Vazyme Biotech Co., Ltd., Nanjing, China). PCR products were quantified using the Quant-iT PicoGreen dsDNA Assay Kit (Thermo Fisher Scientific, Waltham, MA, USA) and Flx800 Microplate Reader (BioTek, Winooski, VT, USA). Sequencing libraries were generated using TruSeq Nano DNA LT Library Prep Kit from Illumina (San Diego, CA, USA), and high-throughput sequencing was performed as the final step.

### 2.4. Enzyme-Linked Immunosorbent Assay for Fecal Inflammatory Markers

After mixing feces with an equal volume of saline, fecal supernatant samples were collected for further analysis. The concentrations of inflammatory markers such as fecal calprotectin (FC) and lactoferrin (LF) were determined in the supernatant according to the manufacturer’s instructions; the cat CALP ELISA kit (ANG-E71001C, Nanjing Jiancheng Bioengineering Institute, Nanjing, China) and cat LTF ELSA kit (ANG-E71005C, Nanjing Jiancheng Bioengineering Institute, Nanjing, China) were then used for the detection procedures.

### 2.5. Fecal Metabolomics Analysis

Short-chain fatty acid concentrations were measured via gas chromatography according to a method described in a previous study [19].

To evaluate fecal metabolomics, fecal samples from 12 healthy cats from day 28 were used. Frozen fecal samples were thawed at 4 °C, and an appropriate amount of sample was transferred into 2 mL centrifuge tubes. A total of 400 μL methanol was added to centrifuge tubes for homogenization. The samples were then centrifuged at 12,000 rpm and 4 °C for 10 min. All supernatant was transferred to new centrifuge tubes, concentrated and dried. The samples were redissolved with 150 μL of a 2-chloro-l-phenylalanine (4 ppm) solution prepared with 80% methanol in water, and the supernatant was filtered using a 0.22 μm membrane for LC-MS detection. Fecal untargeted metabolomic analysis was performed using the Vanquish UHPLC System (Thermo Fisher Scientific, Waltham, MA, USA) and Thermo Q Exactive Focus (Thermo Fisher Scientific, Waltham, MA, USA) and conducted with reference to previous methods [20,21]. An ACQUITY UPLC^®^ HSS T3 (2.1 × 100 mm, 1.8 µm) (Waters, Milford, MA, USA) column was used with a flow rate of 0.3 mL/min, a column temperature of 40 °C and an injection volume of 2 µL. The elution in gradient mode was set to 8% B (0–1 min), 8–98% B (1–8 min), 98% B (8–10 min), 98–8% B (10–10.1 min) and 8% B (10.1–12 min). For LC-ESI (+)-MS analysis, the mobile phases consisted of (B2) 0.1% formic acid in acetonitrile (w/y) and (A2) 0.1% formic acid in water (*v*/*v*). The elution in gradient mode was set to 8% B2 (0–1 min), 8%-98% B2 (1–8 min), 98% B2 (8–10 min), 98–8% B2 (10–10.1 min) and 8% B2 (10.1–12 min). For LC-ESI (-)-MS analysis, the analytes were dissolved in (B3) acetonitrile and (A3) ammonium formate (5 mM). The elution in gradient mode was set as 8% B3 (0–1 min), 8–98% B3 (1–8 min), 98% B3 (8–10 min), 98–8% B3 (10–10.1 min) and 8% B3 (10.1–12 min). The parameters for mass spectrometric detection were set as follows: sheath gas pressure, 40 arbs; aux gas flow, 10 arbs; spray voltage, 3.50 kV and −2.50 kV for ESI (+) and ESI (−), respectively; capillary temperature, 325 °C; MS1 range, *m*/*z* 100–1000; MS1 resolving power, 70,000 FWHM; number of data = dependent scans per cycle, 3; MS/MS resolving power, 17,500 FWHM; normalized collision energy, 30 eV; dynamic exclusion time, automatic.

### 2.6. Fecal Supernatant Antioxidant Capacity Analysis

The levels of total antioxidant capacity (T-AOC), superoxide dismutase (SOD) and malondialdehyde (MDA) in the fecal supernatants on day 7, 14 and 28 were measured using T-AOC (YH1248), SOD (YH1012) and MDA (YH1218) commercial assay kits (Angle Gene, Nanjing, China), respectively.

### 2.7. Statistical Analysis

Data were analyzed using GraphPad Prism 9.3.1. An independent sample *t*-test was used to test the significance of the short-chain fatty acid data, and repeated measures ANOVA was used to assess the significance of the differences in other variables between the two groups; multiple tests were performed using the Bonferroni correction. All results were presented as the mean ± the standard error of the mean (SEM). The difference between the two means was considered statistically significant when *p* < 0.05. All statistics were performed using GraphPad Prism 9.3.1.

Raw data processing for LC-MS was referenced to previous methods but with slight differences [22]. The main difference was that the metabolites were identified using accuracy mass and MS/MS data, which were matched with HMDB (http://www.hmdb.ca, accessed on 3 June 2023), massbank (http://www.massbank.jp/, accessed on 3 June 2023), KEGG (https://www.genome.jp/kegg/, accessed on 3 June 2023), LipidMaps (http://www.lipidmaps.org, accessed on 3 June 2023), mzcloud (https:/www.mzcloud.org, accessed on 3 June 2023) and the metabolite database built by Panomix Biomedical Tech Co., Ltd. (Suzhou, China).

## 3. Results

### 3.1. Growth Perfromence

**Figure 1:**
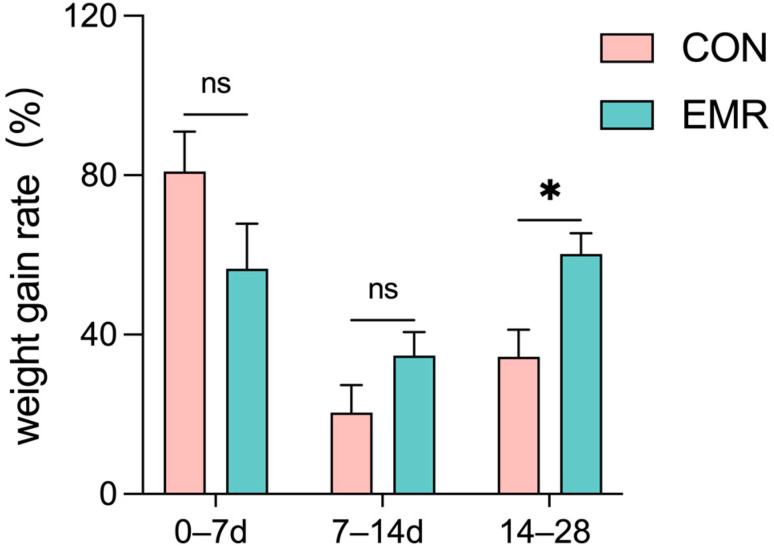
The effect of experimental milk replacer on the weight gain rate of British shorthair kittens. Data on body weight changes in kittens from 0–7 days, 7–14 days and 14–28 days during the trial period were analyzed. Data are presented as mean ± SEM. * *p* < 0.05; ns, *p* > 0.05.

All kittens remained healthy throughout the experiment. As shown in Figure 1, the CON group had a higher rate of weight gain on days 0–7, but there was no significant difference. However, on days 14–28, the EMR group had a significantly higher weight gain rate than the CON group (*p* < 0.05). There was no difference in the weight growth rate between the two groups on days 7–14. Table 4 shows the BW of kittens at the corresponding times.

### 3.2. Analysis of Microbiota Composition

**Figure 2:**
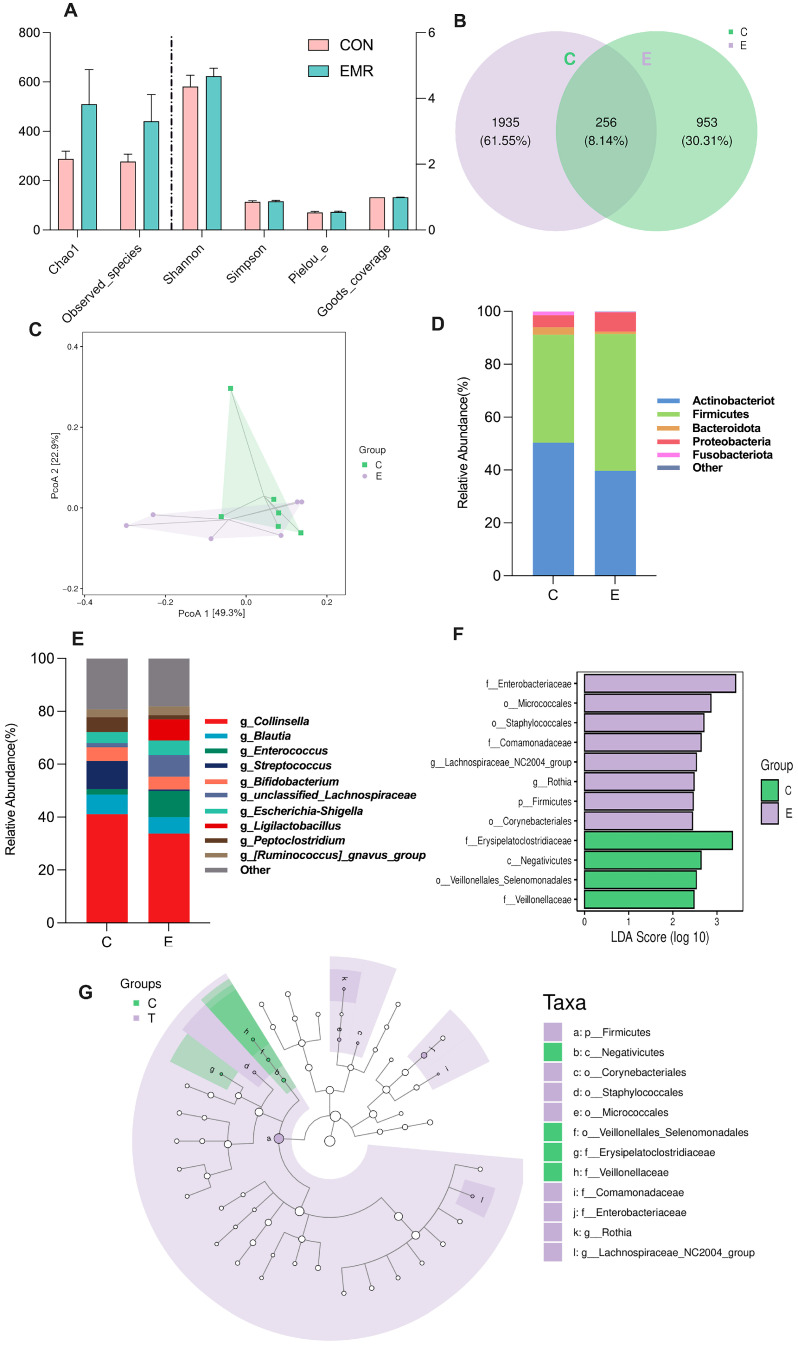
Effects of feeding experimental milk replacer on intestinal microbiome. (**A**) The diversity of the intestinal microbiome in CON and EMR groups (the bars located to the left of the dashed line correspond to the left y-axis, while the bars on the right of the dashed line correspond to the right y-axis). (**B**) OTU Venn diagram. (**C**) Principal coordinate analysis of the intestinal microbiome in CON and EMR groups. (**D**) Effects of feeding experimental milk replacer on the phylum-level composition. (**E**) Effects of feeding experimental milk replacer on the genus-level composition (the legend shows the top 10 genera in terms of abundance). (**F**,**G**) LDA score plot generated from LEfSe of 16S rRNA gene amplification sequencing data (LDA score > 2, *p* < 0.05) and Taxonomic cladogram. Green indicates enriched taxa in the CON group. Purple indicates enriched taxa in the EMR group. Each circle’s size is proportional to the taxon’s abundance. Data are presented as mean ± SEM; C, a control group; E, experimental milk replacer feeding group.

In the alpha diversity analysis of microorganisms, the chao 1 and observed species index represent species abundance and the Shannon and Simpson index are diversity indices. The Pielou’s evenness and Good’s coverage index indicate species evenness and coverage, respectively. No difference was found in the alpha diversity analysis (Figure 2A).

As the Venn diagram shows, the number of operational taxonomic units (OTUs) in the control group was 953, and the number of OTUs in the EMR group was 1935, with a total of 256 OTUs in common between the two groups (Figure 2B). The results from PcoA (Figure 2C) show that the percentage of variation explained by PcoA 1 and PcoA 2 are 49.3% and 22.9, respectively. In the CON group, there were five repetitions that were relatively concentrated, while the EMR group was dispersed in clusters along two directions (Figure 2C).

Furthermore, at the phylum level (Figure 2D), the CON and EMR groups were mainly composed of Firmicutes (CON: 40.9%, EMR: 51.8%), Actinobacteriota (CON: 50.3%, EMR: 39.6) and Proteobacteria (CON: 4.5%, EMR: 7.3%), followed by Bacteroidota (CON: 2.8%, EMR: 1.0%), Fusobacteriota (CON: 1.4%, EMR: 0.1%) and other bacterial groups. There was no significant difference in the relative abundance of microorganism at the phylum level between the CON and EMR groups. At the genus level (Figure 2E), the most dominant genus was *Collinsella* in both groups (CON: 41.1%, EMR: 38.8%). Additionally, *Streptococcus* (10.7%), *Blautia* (7.4%), *Bifidobacterium* (5.1%), *Escherichia-Shigella* (4.2%), *Bacteroides* (2.5%), *Olsenella* (2.4%), *Enterococcus* (2.1%), *Lachnospiraceae* (1.6%) and *Lachnoclostridium* (1.1%) were the remaining abundant genera (>1%) in the CON group. However, the remaining abundant genera (>1%) in the EMR group were *Enterococcus* (9.9%), *unclassified_Lachnospiraceae* (8.2%), *Ligilactobacillus* (8.0%), *Blautia* (6.2%), *Escherichia-Shigella* (5.5%), *Bifidobacterium* (4.8%), *Pediococcus* (3.3%), *R. gnavus group* (3.3%) and *Sellimonas* (2.3%). There was no significant difference in the relative abundance of the top 10 microbial species between the CON and EMR groups (*p* > 0.05).

Utilizing the LEfSe algorithm, our analysis identified distinct microbial signatures for the CON group and for the EMR group. In the CON group, taxa such as *Erysipelatoclostridiaceae* (LDA score = 3.35, *p* < 0.05) and the members of the *Veillonellaceae* family (LDA score = 2.47, *p* < 0.05) were found to be significantly prominent. Conversely, the EMR group was characterized by Staphylococcales (LDA score = 2.53, *p* < 0.05) and *Lachnospiraceae_NC2004_ group* (LDA score = 2.53, *p* < 0.05), Firmicutes (LDA score = 2.46, *p* < 0.05), *Rothia* (LDA score = 2.48, *p* < 0.05), Corynebacteriales (LDA score = 2.44, *p* < 0.05), Micrococcales (LDA score = 2.86, *p* < 0.05), *Comamonadaceae* (LDA score = 2.64, *p* < 0.05) and *Enterobacteriaceae* (LDA score = 3.42, *p* < 0.05). The phylogenetic tree provides a visual representation of these taxa’s evolutionary relationships (Figure 2F,G).

### 3.3. Fecal-Related Inflammatory Indicators

**Figure 3:**
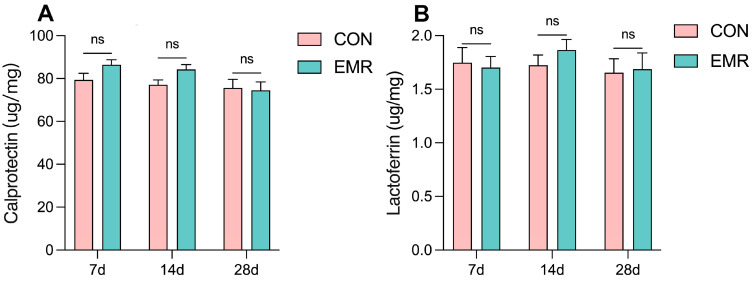
Effect of experimental milk replacer on inflammatory substances in the feces of British Shorthair cats on days 7, 14 and 28 of the trial period. (**A**) Concentrations of calprotectin. (**B**) Concentrations of Lactoferrin. Data are presented as mean ± SEM. ns, *p* > 0.05.

As can be observed in Figure 3, there was no significant difference in the concentrations of lactoferrin and fecal calprotectin between the CON and EMR groups throughout the course of the trial.

### 3.4. Fecal Antioxidant Capacity

**Figure 4:**
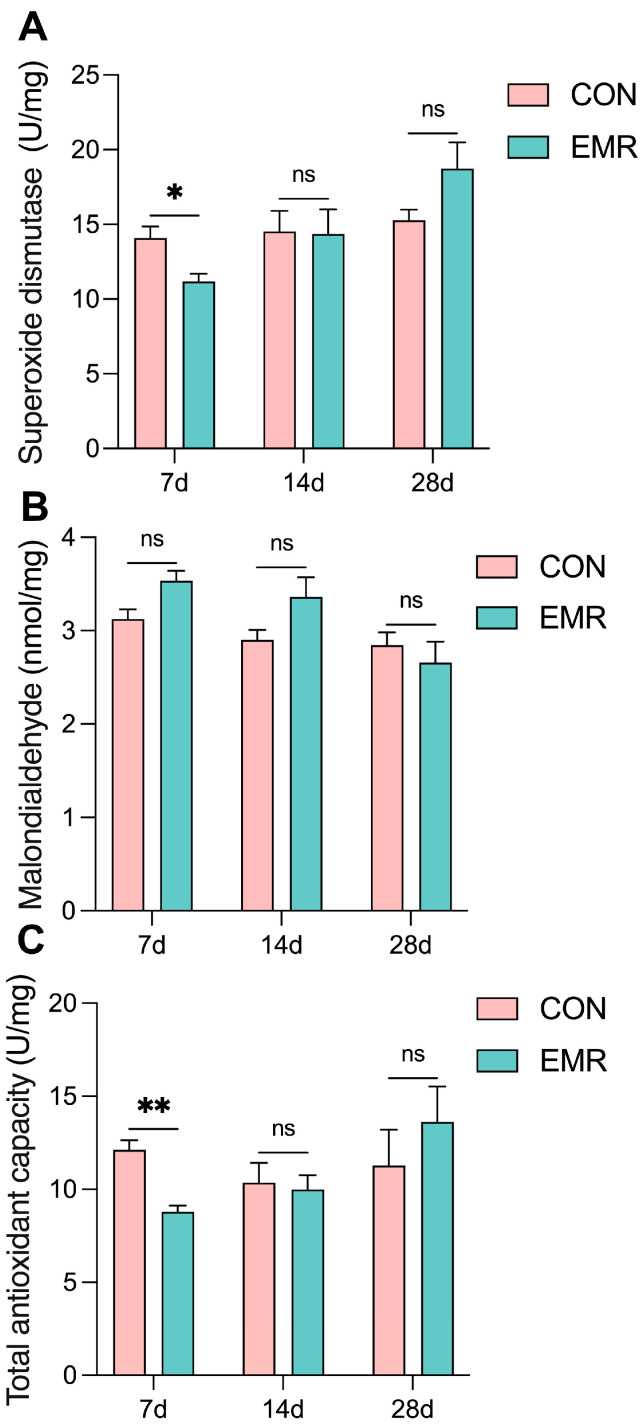
Effect of experimental milk replacer on fecal antioxidant capacity in British shorthair kittens on days 7, 14 and 28. (**A**) Concentrations of superoxide dismutase. (**B**) Concentrations of malondialdehyde. (**C**) Total antioxidant capacity. Data are presented as mean ± SEM. * *p* < 0.05; ** *p* < 0.01; ns, *p* > 0.05.

The CON group showed significantly higher superoxide dismutase (SOD) activity and total antioxidant capacity (T-AOC) than the EMR group on day 7 (*p* < 0.05 and *p* < 0.01, respectively), while there were no significant differences between the CON and EMR groups on days 14 and 28 (Figure 4A,C). On the other hand, there was no significant difference in malondialdehyde (MDA) levels throughout the trial (Figure 4B). Nonetheless, the antioxidant capacity of the EMR group showed a tendency to catch up and surpass that of the CON group throughout the trial period.

### 3.5. Microbiota-Derived SCFA Production

**Figure 5:**
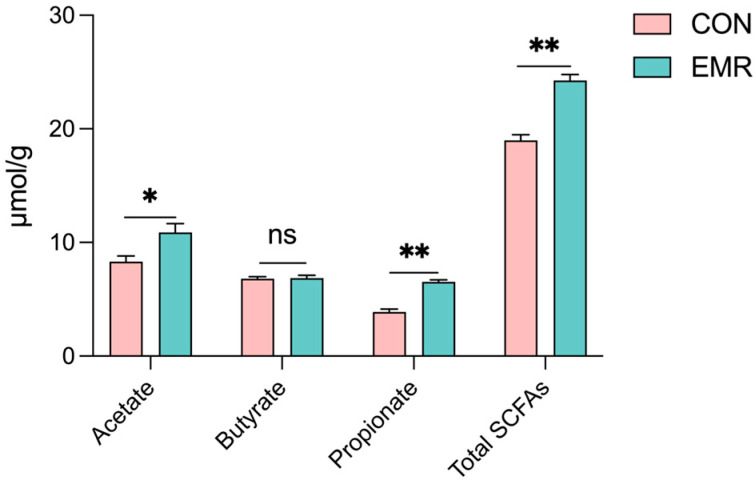
Effects of experimental milk replacer on fecal fermentation metabolites in British shorthair kittens. Data are presented as mean ± SEM. * *p* < 0.05; ** *p* < 0.01; ns, *p* > 0.05.

As shown in Figure 5, acetate acid (*p* < 0.05), propionate acid and total SCFAs concentrations were elevated in the EMR group, with highly significant increases in propionate acid (*p* < 0.01) and total SCFAs (*p* < 0.01). There was no significant difference between the two groups in terms of butyrate acid.

### 3.6. Analysis of Fecal Metabolites

**Figure 6:**
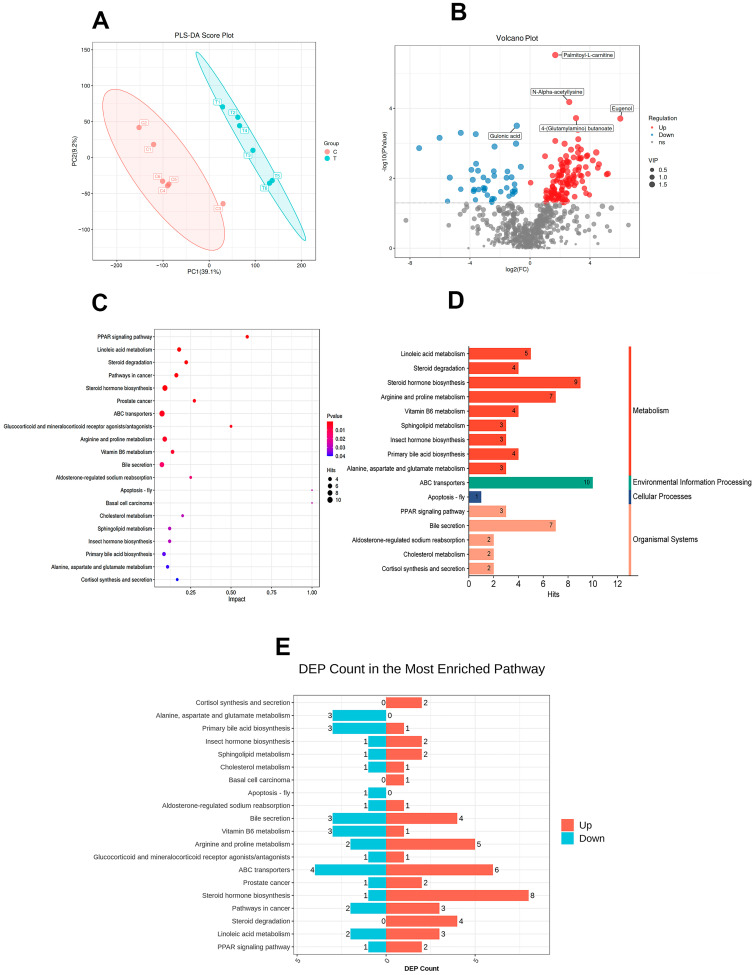
Effect of experimental milk replacer on fecal metabolites in British shorthair kittens. (**A**) PLS-DA models of fecal metabolites in CON and EMR groups (PLS-DA models: R2Y = 0.998 and Q2 = 0.907). (**B**) Volcano plot of differently expressed fecal metabolites. The dashed horizontal line signals statistical significance threshold (*p* ≤ 0.05). (**C**) Metabolic pathways influencing factor bubble diagram. (**D**) Classification of significantly enriched KEGG pathways of differently expressed metabolites (pathways that do not demonstrate relevance to the classification of human disease and drug development). (**E**) Statistical map of the number of differentially enriched metabolic pathways.

The PLS-DA model (Figure 6A) demonstrated that both the CON group and the EMR group could be separated clearly (PLS-DA models: R2Y = 0.998 and Q2 = 0.907). After the secondary differential metabolite count, a total of 167 differential metabolites were identified (VIP > 1.0 and *p* < 0.05), of which 125 were up-regulated differential metabolites and 42 were down-regulated differential metabolites. Palmitoyl-L-carnitine, N-alpha-acetyllysine, eugenol, 4-(glutamylamino) butanoate and gulonic acid were the five differential metabolites with the most significant differences (Figure 6B).

In the KEGG pathway analysis, 20 pathways were identified as significant. Figure 6C illustrates all differential pathways with *p*-values < 0.04. Pathways exhibiting highly significant differences (*p* < 0.01) were the PPAR signaling pathway, linoleic acid metabolism, steroid degradation, pathways in prostate and other cancers, steroid hormone biosynthesis, ABC transporters, glucocorticoid and mineralocorticoid receptor agonists/antagonists, arginine and proline metabolism, and vitamin B6 metabolism. According to the KEGG pathway database, all pathways can be grouped into six categories. In addition to pathways related to human disease and drug development, the highest number of pathways belong to metabolism, followed by organismal systems, environmental information processing and cellular processes, which are represented in the lowest numbers (Figure 6D). Figure 6E shows the number of up-regulated differential metabolites and down-regulated differential metabolites participating in these differential pathways.

## 4. Discussion

Breast milk is widely acknowledged as the optimal source of neonatal nutrition [23]. In the initial phase of our study, maternal milk provided kittens with rich nutrition and immune defense. On the other hand, the stress of weaning and separation from the mother reduced the growth rate of the EMR group [24]. These resulted in higher early growth rates in kittens fed with maternal milk. However, during the later stages of the trial, the weight growth rate in the EMR group was significantly higher than that of thematernal milk group. The nutrient profile of feline maternal milk is subject to fluctuations influenced by the lactation stage, environmental conditions and the queen’s nutritional state, among other variables [11,25,26]. As lactation progresses, fluctuations in maternal nutritional reserves can lead to instability in the nutritional value of queen’s milk. By contrast, EMR is relatively stable nutritionally and can support kitten growth. At this stage, kittens have also adapted to the separation from their mother and the change in diet. This is consistent with the results of a previous study on milk replacer feeding [27]. However, rapid weight gain does not imply an exclusively positive effect, and milk replacers are still only used as an adjunctive lactation option, with the potential physiological effects on kittens still to be further investigated.

The gastrointestinal tract represents a complex micro-ecosystem modulated by dietary constituents and host-derived inputs, which collectively orchestrate the gut microbiota’s richness, diversity and functional output [28,29]. Our investigation revealed no discrepancy in alpha diversity indices between the CON and EMR group, suggesting comparable microbial community richness and uniformity. Nonetheless, discrepancies in beta diversity, as evidenced by OTU Venn diagrams and principal coordinates analysis (PCoA) plots, were attributable to EMR. At the phylum level, the dominant gut microbiota, including Firmicutes, Actinobacteria, Bacteroidetes, Proteobacteria and Fusobacteria remained statistically analogous, consistent with previous studies of cat gut flora [30]. At the genus level, a relative increase in *Enterococcus*, *unclassified-Lachnospiraceae* and *Ligilactobacillus* was observed in the EMR group, contrasted by a decrease in *Streptococcus*. Integrative analysis utilizing taxonomic branching diagrams and linear discriminant analysis (LDA) effect size (LEfSe) scoring delineated 12 distinct bacterial taxa, notably within the Veillonellaceae, Erysipelatoclostridiaceae, *Lachnospiraceae_NC2004_group*, and *Rothia* clades. The changes in the gut microbiota may be due to the addition of fructo-oligo saccharides (FOS), which are considered significant prebiotics that can modulate gut microbiota, thereby affecting the gut immune system, inflammatory responses and oxidative stress, ultimately preventing gut damage and inflammation [31]. Previous research has indicated that FOS supplementation increased the abundance of *Ligilacillus* in a human colonic fermentation model [32]. A prebiotic complex containing FOS improved DSS-induced colitis by altering the intestinal microbiome, increasing the abundance of *Ligilactobacillus*, *Bifidobacterium* and *Limosilactobacillus*, and exerting anti-inflammatory effects [33]. Among the differential flora, genomic inquiry underscores *Lachnospiraceae*’s adeptness in polysaccharide catabolism, starch and saccharide hydrolysis and subsequent short-chain fatty acid (SCFA) biosynthesis, including propionate [34,35,36]. This is consistent with our measurements of fecal SCFAs (Figure 5). *Rothia* is a bacterium that primarily produces butyrate [37]. Yet there is no significant difference in butyric acid content in Figure 5, due to the fact that, although the high-latitude Lefse analyses showed differences in *Rothia*, the relative abundance of *Rothia* was still <1% and could not affect butyric acid production. While EMR did not perturb the overarching gut microbial structure, it did modulate the genus-level composition, thereby promoting the colonization of SCFA production-related bacteria in kittens [38].

The extent of intestinal inflammation in felines can be gauged through assays of fecal calprotectin (FC) and lactoferrin (LF). Previous studies have shown that FC can be used to some extent as a biomarker for feline chronic inflammatory enteropathies [39,40]. LF is an immune-competent component of colostrum, and supplementation with LF can boost immunity and antioxidant capacity [8,41]. Our data indicated parity in LF and FC levels between the CON and EMR groups. This may be due to the fact that all of our kittens underwent a week-long period of acquiring immunity and had developed a certain immune base; it follows that no differences were found. On the other hand, superoxide dismutase (SOD) activity and total antioxidant capacity (T-AOC) was diminished in the EMR group on the seventh day compared to the CON group (Figure 4). SOD is a marker of antioxidant protection, whereas MDA is a marker of serum lipid peroxidation [42]. These biomarkers—SOD, MDA, and T-AOC—serve as indices of oxidative stress, implicating an increased oxidative burden in the EMR group during this phase. Subsequent to this period, both antioxidant and inflammatory markers normalized, a phenomenon that can be explained by the elevated short-chain fatty acids (SCFAs) observed in the EMR group. SCFAs, pivotal microbial metabolites, furnish energy for colonic and ileal epithelia and modulate immunity via cell-surface fatty acid receptors and G protein-coupled receptors (GPCRs) such as GPR41, GPR43 and GPR109A, influencing T-cell and B-cell differentiation and adaptive immunity development [43,44]. SCFAs mitigate inflammation through histone deacetylase (HDAC) inhibition, modulating macrophage activity, T-cell factor expression and effector T-cell differentiation [45]. Additionally, a previous study found that SCFAs may bolster antioxidant defenses in mice by inducing the expression of nuclear factor erythroid 2-related factor 2 (Nrf2) or heme oxygenase-1 (HO-1) [46]. These results suggest that EMR alters the composition of the intestinal microbiome, which in turn promotes SCFA production, leading to enhanced antioxidant capabilities in kittens [47].

We used off-target metabolic analysis to cover as much of the metabolic space as possible for metabolic detection in kitten feces [48]. Through the analysis of KEGG pathwayenrichment of differential metabolites, we discerned 20 implicated pathways, with 18 exhibiting upregulated involvement of differential metabolites. These pathways are primarily related to nutrient metabolism, particularly lipid metabolism, including linoleic acid metabolism, steroid degradation, steroid hormone biosynthesis, sphingolipid metabolism and primary bile acid biosynthesis. Amino acid metabolism was also involved to a lesser extent. Additionally, ABC transporters, which facilitate the translocation of sterols, lipids and lipid-like molecules, including cholesterol and sphingolipids, play a pivotal role in the reverse transport of cholesterol from peripheral tissues [49]. Peroxisome proliferator-activated receptors (PPARs) are instrumental in both lipid and glucose metabolism [50]. Cholesterol, serving as a precursor for various hormones such as bile acids and cortisol, is central to cholesterol and bile acid metabolism, which are vital for lipid digestion and absorption [51]. The observed metabolic patterns can be attributed to the presence of various ingredients that supplemented polyunsaturated fatty acids (PUFAs) in powdered milk formulations, such as sunflower seed oil powder, flaxseed oil powder and arachidonic acids, which are added to the formula. PUFAs exert influence on hepatic lipid metabolism and physiological responses across diverse organ systems [52]. Moderate intake of PUFAs has been shown to decrease total blood cholesterol and low-density lipoprotein (LDL)-cholesterol levels [53]. Furthermore, PUFAs may induce the transcription of the LXRα gene, potentially via PPAR activation, which in turn enhances CYP7 activity, facilitating cholesterol catabolism for bile synthesis [52,54]. Previous studies have also shown a reduction in circulating cholesterol and triglycerides through dietary PUFA supplementation [55]. These findings highlight the potential benefits of including PUFAs in kitten milk replacers, suggesting a positive impact on metabolic health, but further correlational studies are still needed to validate whether alterations in metabolism may have other potential effects.

The focus of this study was the effects of EMR on gut microbiota composition and metabolic profiles. Our findings suggest that EMR can modulate the composition and metabolism of the intestinal microbiome, improve the growth performance and antioxidant capacity of weaned kittens and regulate nutrient metabolism, especially lipid metabolic pathways. However, the role played by the bioactive components of EMR in the growth and development of weaned kittens remains to be further explored, including the combined effects among bioactive components. The results of this test were also limited by factors such as the number of test kittens, individual differences and the test environment, which need to be improved in the future. In addition, this study strictly adhered to the principles of animal welfare, which imposed some limitations. Kittens produce little feces in the early stages of life, and protecting the test animals from stress made it impossible to collect these and other samples as required; as a result, we could not track the physiological conditions of the kittens from the beginning of the experiment. This will need to be addressed in the future.

## 5. Conclusions

In conclusion, kittens fed with EMR exhibited a faster growth rate during the late lactation period. Compared to nursing, the intestinal microbiome of kittens fed EMR was altered, with an increase in the relative abundance of *Enterococcus*, *Lachnospiraceae* and *Ligilactobacillus*. EMR also affects the production of SCFAs and the antioxidant capacity of kittens. In addition, EMR activates metabolic pathways related to lipid metabolism. However, this study had several limitations. The results were limited to EMR containing specific components and were constrained by factors such as the number of test kittens, individual differences and the experimental environment. These factors present areas for future improvement. This study provides insights into the effects of kitten milk replacer on the gut microbiota and metabolic profile of kittens. The findings indicate the potential impact of EMR in kitten nutrition and pave the way for further in-depth studies to optimize kitten milk replacer formulations.

## Figures and Tables

**Table 1:** Dietary composition and nutritional content of kitten formula milk powder ^1^.

Dietary Composition	%	Nutrient Content	%
Goat milk power	36.00	Moisture	3.50
Whey protein concentrate	27.72	Crude protein	33.00
Cream powder	20.00	Crude fat	39.00
Cheese powder	4.00	Ash	4.00
Sunflower seed oil powder	6.00	Calcium	1.10
Flaxseed oil powder	1.00	Phosphorus	0.80
Palmitic triglyceride	1.00		
Calcium hydrogen phophate	1.10		
Tricalcium phosphate	0.60		
Fructooligosaccharide	1.00		
L-arginine	0.60		
Fish oil powder	0.35		
Taurine	0.25		
Colostrum Powder	0.10		
Arachidonic acid	0.10		
Salt	0.10		
Lactoferrin	0.03		
Mineral complexes and vitamins ^2^	0.05		

^1^ Sourced from labeling instructions and related information provided by the manufacturer. ^2^ Mineral complexes and vitamins per kilogram of milk powder: vitamin A (14,500 IU), vitamin D_3_ (1000 IU), vitamin E (156 IU), vitamin B_1_ (32.0 mg), vitamin B_2_ (30.0 mg), vitamin B_3_ (120 mg), vitamin B_5_ (88.0 mg), vitamin B_6_ (13.0 mg), vitamin B_12_ (0.20 mg), Fe (FeSO_4_) 100 mg, Cu (CuSO_4_) 7.00 mg, Co (CoSO_4_) 1.00 mg, I (CaI_2_) 20.0 mg, Mn (MnSO_4_) 20.0 mg, Zn (ZnSO_4_) 68.0 mg and Se (Na_2_SeO_3_) 0.50 mg.

**Table 2:** Recommended daily intake of kitten formula milk powder ^1^.

Kitten Age/Week	Number of Meals/Days	mL of Milk per Meal
1	7	2–4
2	6	5–10
3	5	10–15
4	5	10–15
5	4	10–15

^1^ Sourced from labeling instructions and related information provided by the manufacturer.

**Table 3:** Grouping of kittens after a one-week period of acquired immunization.

Litters	CON	EMR	Male:Female
Litter 1	N = 2	N = 2	3:1
Litter 2	N = 2	N = 2	1:1
Litter 3	N = 2	N = 2	1:3
Total	N = 6	N = 6	1:1

**Table 4:** Kittens’ body weight.

Experimental Time (Day)	CON	EMR
	BW (g)	BW (g)
0	138.33 ± 6.78	139.87 ± 10.92
7	249.50 ± 15.67	222.72 ± 24.26
14	297.00 ± 14.76	298.38 ± 30.99
28	396.83 ± 20.78	470.72 ± 40.49

Values are presented as mean ± SEM, *n* = 6; CON = control group; EMR = group fed with an experimental milk replacer.

## Data Availability

The data underlying this article will be shared upon reasonable request to the corresponding author. The data are not publicly available, because the kitten milk replacer product is under development and the subject of a patent application.
